# Cataract Surgery in the Glaucoma Patient: Beyond Intraocular Pressure

**DOI:** 10.5005/jp-journals-10008-1144

**Published:** 2013-09-06

**Authors:** Kuldev Singh

**Affiliations:** Professor, DDepartment of Ophthalmology, Stanford University, Medical Center, Byers Eye Institute at Stanford, 2452 Watson Court, MC 5353, Palo Alto, CA 94303, USA

**Keywords:** Cataract, Glaucoma, Intraocular pressure.

## Abstract

**How to cite this article:** Singh K. Cataract Surgery in the Glaucoma Patient: Beyond Intraocular Pressure. J Current Glau Prac 2013;7(3):97-98.

Over the past decade, an ever increasing body of evidence has shown that modern cataract surgery via phacoemulsification should be considered an intraocular pressure (IOP) lowering procedure.^1-3^ This confirmation of a long held belief has led to the consideration of earlier cataract surgery, for purposes of IOP reduction, in those with concomitant glaucoma and cataract. One should be aware, however, that this IOP lowering effect with cataract surgery is unpredictable with some getting a substantial reduction while others show little change, or even an increase in IOP that may be harmful to the optic nerve in the postoperative period.

The average magnitude of IOP lowering with cataract surgery is not enough to consider it a worthwhile stand alone glaucoma procedure in some with even mild to moderate optic nerve damage which has led to the proliferation of minimally invasive glaucoma procedures that are combined with cataract surgery. Yet the most important reasons for performing early cataract surgery in those with glaucoma may have little to do with IOP lowering.

Glaucoma management in those who are pseudophakic is, in many ways, easier than it is for phakic individuals. Even prior to the degree of cataract maturity traditionally required to pursue surgical removal, the impact of the lens on the diagnosis of disease, as well as the measurement of the rate of progression, can be problematic. Both structural and functional testing of the optic nerve may be infuenced by lens changes and every experienced glaucoma practitioner is aware of the difficulty in assessing presumed progressive visual afield loss related to glaucoma that is confounded by a cataractous lens.

A major advantage of early cataract surgery in those with glaucoma is that all future glaucoma treatment options are preserved and some may actually be improved. Clear corneal temporal phacoemulsification spares the superior conjunctiva making future trabeculectomy no more complex or likely to fail than in phakic eyes. There may be additional safety benefits with trabeculectomy in pseudophakic eyes as many postoperative problems are related to the lens. While studies have not adequately assessed this hypothesis, shallow and fat anterior chambers are perhaps less common in pseudophakic than phakic eyes for a variety of postulated reasons. In addition, pseudophakic trabeculectomy may be less likely to necessitate an iridectomy to prevent postoperative iris prolapse. Pseudophakia is also associated with increased anterior chamber depth making subsequent drainage device implantation easier and safer. In addition to preserving all glaucoma surgical options, cataract surgery may allow for the subsequent use of effective miotic medications, a class of drugs often associated with intolerable ocular side effects in phakic individuals.

One of the most worrisome aspects of glaucoma care is performing a cataract procedure in an eye with a functioning fltration bleb. Despite our best efforts including the use of perioperative antifibrotics and steroids, many such blebs will fail following cataract surgery. Others will function less effectively resulting in a greater dependence on medications and an accelerated course to failure necessitating a glaucoma reoperation. While this issue of deceased fltration following cataract surgery has not been well studied in the case of glaucoma drainage devices, one can reasonably hypothesize that the infammation and subsequent scarring from cataract surgery likely has an adverse impact on IOP in such eyes as well. All of these potential problems are, of course, obviated if the cataract operation is performed prior to the glaucoma procedure.

It is important to keep in mind; however, that cataract surgery is not a panacea when it comes to glaucoma management. Patients may have dangerous elevation of IOP following cataract surgery which sometimes needs to be addressed urgently to minimize permanent vision loss and, unfortunately, there are few preoperative predictors of which individuals will follow such a course. The practitioner has to be prepared to urgently perform glaucoma surgery that may have been considered electively, sometimes in combination with cataract surgery, prior to the decision to pursue cataract surgery alone. This risk, if dealt with appropriately, is a small price to pay for the potential benefit of avoiding, or at least delaying, the need for a glaucoma fltration operation, particularly in those with mild to moderate disease. The risk of postoperative vision loss related to IOP spikes is too high, however, to consider cataract surgery alone in those with moderate to severe glaucomatous optic nerve damage.

Such individuals will require glaucoma fltration surgery as the initial surgical procedure, sometimes combined with cataract surgery.

The evolution of cataract surgery over the past half century has had a positive impact on the care of open angle and angle closure glaucoma worldwide. The IOP lowering with phacoemulsification is only part of the story and the other benefits are, in some ways, more compelling when advocating for this procedure in the glaucoma patient.
